# Surgical quality assessment of critical view of safety in 283 laparoscopic cholecystectomy videos by surgical residents and surgeons

**DOI:** 10.1007/s00464-024-10873-0

**Published:** 2024-05-20

**Authors:** Alexander A. J. Grüter, Freek Daams, Hendrik J. Bonjer, Peter van Duijvendijk, Jurriaan B. Tuynman, Video-Based Surgical Quality Assessment Collaborators, Anneke Jilesen, Björn Blomberg, Bob Berndsen, Carlijn de Betue, Daan Henneman, Didi Sloothaak, Eelco Wassenaar, Emma Bruns, Emma Westerduin, Ernst-Jan van Nieuwenhoven, Franceline Frans, Frank Hoexum, Fred Prakken, Gijs Musters, Hamid Jalalzadeh, Harm Ebben, Harm Willem Palamba, Jasper Atema, Jelle Posthuma, Johan Dikken, Johannes Govaert, Jony van Hilst, Joost ten Brinke, Jose Volders, Kevin de Leur, Klaas Govaert, Leonie van der Werf, Linde Busweiler, Marco Goessens, Marieke Bolster-van Eenennaam, Martijn van Dorp, Ninos Ayez, Noor Karthaus, Patrick Moerbeek, Paul Johannesma, Robert-Jan Coelen, Robin Blok, Roel Bakx, Sander Mekke, Sarah Gans, Stefan van Dijk, Stijn van der Ploeg, Thomas Poels, Usha Coblijn, Victor Alberts, Viole Weeda, Wijnand Alberda, Willem Lastdrager, Yama Issa

**Affiliations:** 1 Department of Surgery, Amsterdam UMC Location Vrije Universiteit Amsterdam, De Boelelaan 1117, 1081 HV Amsterdam, The Netherlands; 2 Cancer Center Amsterdam, Treatment and Quality of Life, Amsterdam, The Netherlands; 3 Department of Surgery, Gelre Hospitals, Albert Schweitzerlaan 31, Apeldoorn, The Netherlands

**Keywords:** Surgical quality assessment (SQA), Laparoscopic cholecystectomy, Video-based assessment, Training

## Abstract

**Introduction:**

Surgical quality assessment has improved the efficacy and efficiency of surgical training and has the potential to optimize the surgical learning curve. In laparoscopic cholecystectomy (LC), the critical view of safety (CVS) can be assessed with a 6-point competency assessment tool (CAT), a task commonly performed by experienced surgeons. The aim of this study is to determine the capability of surgical residents to perform this assessment.

**Methods:**

Both surgeons and surgical residents assessed unedited LC videos using a 6-point CVS, a CAT, using an online video assessment platform. The CAT consists of the following three criteria: 1. clearance of hepatocystic triangle, 2. cystic plate, and 3. two structures connect to the gallbladder, with a maximum of 2 points available for each criterion. A higher score indicates superior surgical performance. The intraclass correlation coefficient (ICC) was employed to assess the inter-rater reliability between surgeons and surgical residents.

**Results:**

In total, 283 LC videos were assessed by 19 surgeons and 31 surgical residents. The overall ICC for all criteria was 0.628. Specifically, the ICC scores were 0.504 for criterion 1, 0.639 for criterion 2, and 0.719 for the criterion involving the two structures connected to the gallbladder. Consequently, only the criterion regarding clearance of the hepatocystic triangle exhibited fair agreement, whereas the other two criteria, as well as the overall scores, demonstrated good agreement. In 71% of cases, both surgeons and surgical residents scored a total score either ranging from 0 to 4 or from 5 to 6.

**Conclusion:**

Compared to the gold standard, i.e., the surgeons’ assessments, surgical residents are equally skilled at assessing critical view of safety (CVS) in laparoscopic cholecystectomy (LC) videos. By incorporating video-based assessments of surgical procedures into their training, residents could potentially enhance their learning pace, which may result in better clinical outcomes.

Quality assessment in laparoscopic surgery has shown improvements in effectiveness and efficiency of surgical training, potentially enhancing learning curves and improving outcomes [1]. The surgical technique is presumed to be an important factor in patient outcomes. Increasing evidence indicates that the quality of an operation, measured by surgical quality assessment (SQA) tools, is directly correlated with clinical outcomes [2–5].

Since its introduction in the late 1980s, laparoscopic cholecystectomy (LC) has become the method for removing the gallbladder, overtaking the open surgery technique. This preference is largely due to the reduced postoperative pain and shorter recovery times associated with the minimally invasive approach. [6–8]. However, during the initial learning phase of LC, there is a noted increase in bile duct injuries (BDIs) compared to the traditional open surgery. Previously, the rate of BDI during open cholecystectomy was between 0.1% and 0.2% [9–14]. In comparison, the rate of BDI after LC has stabilized at around 0.5% [9–14]. Such injuries can have serious consequences for patients and the health-care system, often necessitating complex and costly surgical repairs of the bile ducts. 

In the early nineties, Strasberg emphasized the importance of achieving the critical view of safety (CVS) in every LC [15]. The prevalence of bile duct injury in LC is linked to the attainment of CVS. Performing a CVS during dissection of the gallbladder pedicle plays a highly protective role against BDI due to misidentification [16]. Successful attainment of the CVS involves the following three criteria: identification of only two structures entering the gallbladder (cystic duct and cystic artery), clearance of the bottom one-third of the gallbladder from the liver to expose the cystic plate, and clearance of the hepatocystic triangle of all tissues except the cystic artery and cystic duct. The hepatocystic triangle is formed by the cystic duct, the common hepatic duct, and the inferior edge of the liver. Sanford et al. developed a 6-point CVS assessment tool, wherein each criterion is evaluated on a scale from 0 to 2, and a score of 5 or 6 indicates a “favorable” CVS [17].

Currently, the assessment of a CVS is typically conducted by the performing and/or supervising surgeon, while LC procedures are often performed by surgical residents. To optimize the learning curve, there is a need for objective video-based assessments that can be easily integrated into clinical practice. However, these video-based assessments can be time-consuming for senior expert surgeons. Therefore, the primary aim of this study was to determine whether surgical residents can assess CVS as effectively as surgeons. If the assessments of the surgical residents are comparable to those of the senior surgeons, then surgical residents could potentially self-assess in future, resulting in substantial time savings for senior surgeons.

## Methods

**Fig. 1:**
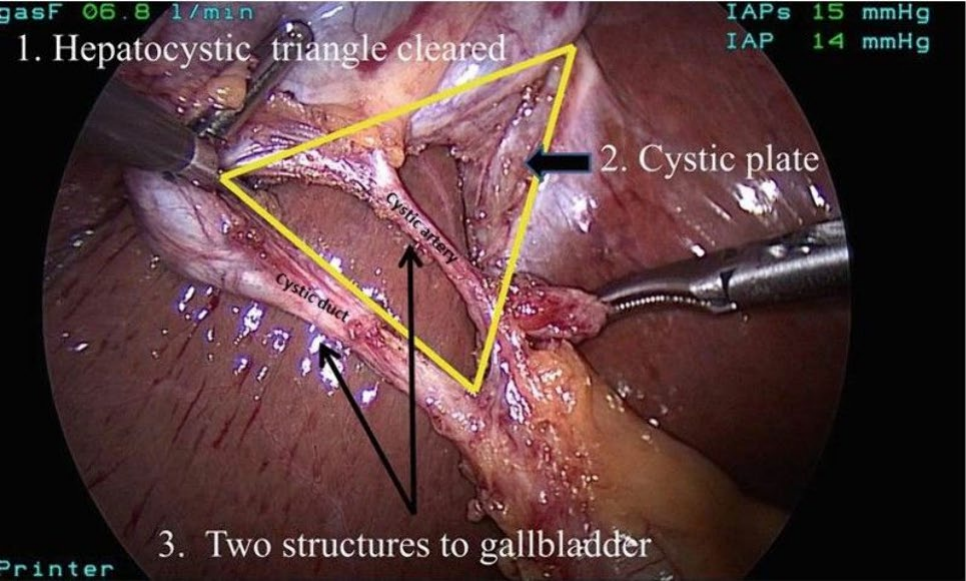
The three criteria defining the critical view of safety (CVS) in laparoscopic cholecystectomy

Prior to the study, an overview was compiled of surgeons and surgical residents known to us who have experience in performing LC. Subsequently, all of them were approached and asked whether they were willing to participate in this study. They were asked to assess the CVS in unedited full-length anonymized LC videos, all of which were performed at Gelre Hospitals. The assignment of videos to reviewers was randomized with the condition that reviewers did not evaluate their own video. Each video underwent assessment by both a surgeon and a surgical resident, utilizing the adapted 6-point CVS assessment tool by Sanford et al. [17]. Each criterion of CVS could receive a score ranging from 0 to 2 points, resulting in a total potential score of 6 points, see Fig. 1 and Table 1. A higher score indicated a better performance of the corresponding aspect. All videos were assessed on an online platform, called SQA, and set up by the Amsterdam Skills Centre (https://asc.amsterdam/sqa-amsterdam/). For each scored video, a small financial compensation was given.

**Table 1:** Critical view of safety (CVS) assessment tool adapted from Sanford et al. [17]

Criterion	Score	Description
1. Clearance of hepatocystic triangle	0	Tissue in triangle obscures the view of cystic structures and cystic plate and does not allow conclusion that there are no other structures in triangle. Or technical issues prevent determination of how well cleared the triangle is
	1	Somewhat less than the whole triangle can be clearly seen or technical issues reduce the ability to see optimally
	2	Hepatocystic triangle is cleared of tissue so that visibility of cystic structures and plate is completely unimpeded, but also so that viewer can be immediately certain than no other structures are in the triangle
2. Cystic plate	0	Cystic plate is not visible due to positioning, light, obstruction of view by instruments, or coverage with clot
	1	Cystic plate is visible but overlapped by other structures so that it is not optimally seen or an insufficient amount of the plate is shown
	2	Cystic plate is immediately clearly visible to approximately its bottom one-third
3. Two structures connect to the gallbladder	0	Due to overlap or technical issues, two separated cystic structures cannot be seen
	1	Two structures can be seen connecting to the gallbladder, but there is some overlap of duct and artery or a technical feature, such as poor lighting or lack of color contrast, that interferes with clarity of determination
	2	Two structures can immediately and clearly be seen connecting to the gallbladder

Multiple reminders were sent to both the surgeons and surgical residents to ensure the assessment of the videos, and only those videos that received double scores were included in the analyses. Videos were also excluded for further analysis if for example the CVS was not visible in that video (for example in the case of conversion before CVS was visible), if the video did not appear to be a surgical procedure, if it involved an operation other than a LC or if the media could not be loaded. Patients undergoing LC for cholecystitis were included, and videos were excluded if the CVS was not adequately assessable, for example, in some cases of subtotal cholecystectomy or retrograde cholecystectomy. This constitutes a retrospective study in which the videos were generated automatically. Due to the absence of identifiable patient information in these videos, IRB approval or written consent from the patients was deemed unnecessary.

### Statistical analysis

The inter-rater reliability between surgeons and surgical residents, both for the overall score and the scores of the three criteria separately, was assessed using the intraclass correlation coefficient (ICC). This analysis employed a two-way random, absolute, and average measurement effect model, with a two-sided significance level of *P* < 0.05. ICC values of < 0.4, > 0.4 and ≤ 0.6, > 0.6 and ≤ 0.8, and > 0.8 were regarded as poor, fair, good, and excellent agreement, respectively, whereas values of ICC of ≤ 0.4 were judged to have poor agreement [18].

In addition, percentages were employed to describe the agreement, and the McNemar test was utilized to assess whether there was a significant difference in scoring either 0–4 or 5–6 between both attending surgeons and surgical residents. A *P* value less than 0.05 was considered statistically significant.

## Results

**Fig. 2:**
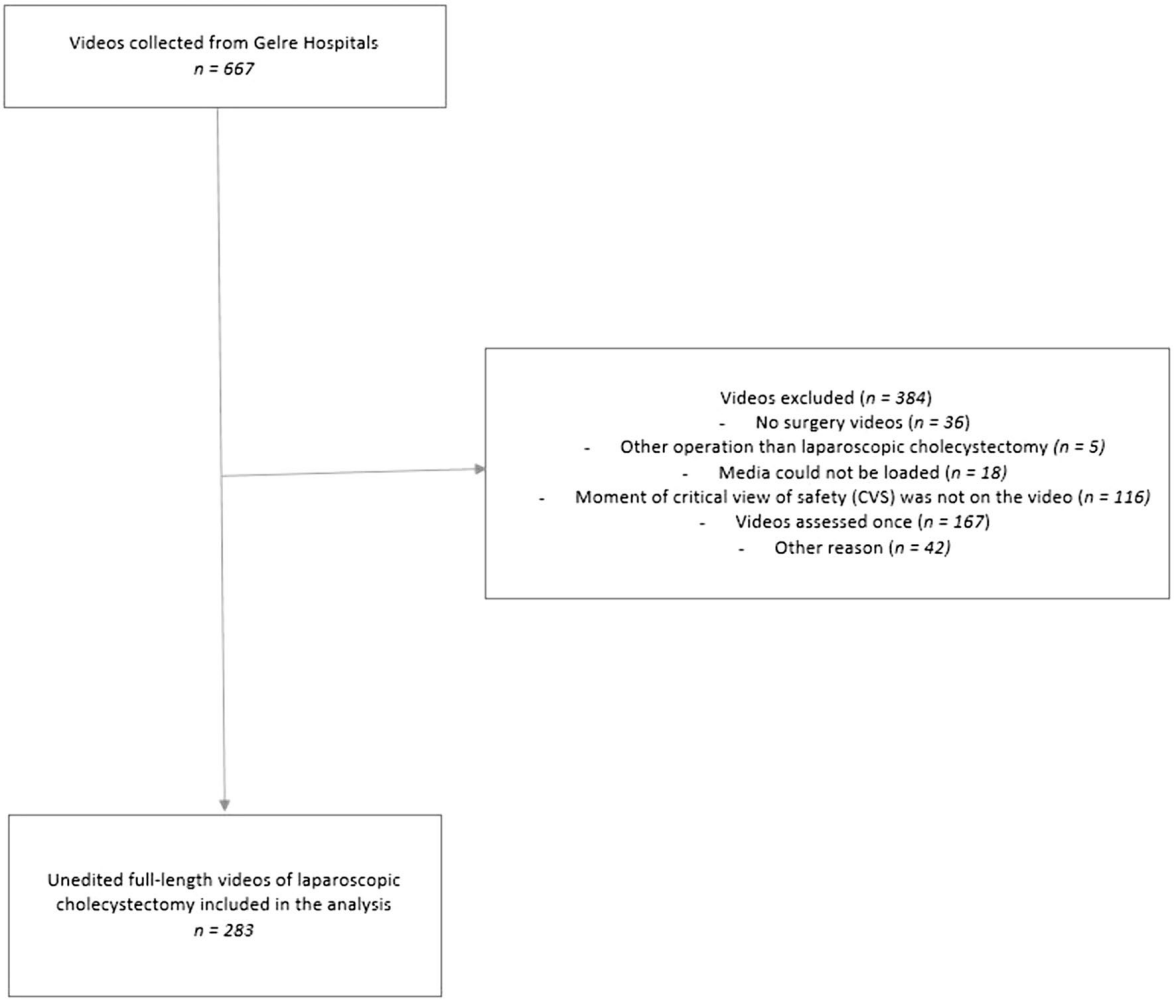
Flow diagram of collection of videos showing reasons why videos were excluded and the actual videos analyzed

A total of 667 videos were retrospectively collected and 283 videos underwent assessment by both a surgeon and a surgical resident, see Fig. 2 for the reasons of exclusion. Thirty-one surgical residents and 19 surgeons participated in this study and assessed the LC videos.

The overall ICC score for all scores together between surgeons and surgical residents was 0.632. Specifically, the ICC for clearance of hepatocystic triangle was 0.508, for cystic plate was 0.641, and for two structures connect to the gallbladder was 0.721, see Table 2. So fair agreement was observed only for the criterion related to the clearance of the hepatocystic triangle and there was good agreement for the other two criteria and for all scores together.

**Table 2:** The total intraclass correlation coefficient (ICC) and the ICC per criterion of the critical view of safety (CVS) assessment tool

Criterion CVS	1. Clearance of hepatocystic triangle	2. Cystic plate	3. Two structures connect to the gallbladder	1–3 (total)
ICC	0.504	0.639	0.719	0.628

In 71.0% of these cases, there was agreement that both the surgeon and the surgical resident had assigned a total score of 0 to 4, or both had given a total score of 5 or 6. In 29% of the cases, there was no agreement between surgical residents and attending surgeons in assigning either a total score of 0–4 or 5–6 to a single video. In 36.6% of these cases, the surgeon scored 0–4, while the surgical resident scored 5 or 6, and in 63.4% of the cases, the surgeon scored 5 or 6, while the surgical resident scored 4 or lower, see Table 3. This difference is statistically significant with a *P* value of 0.02.

**Table 3:** The number of total scores ranging from 0 to 4 and the number of total scores 5 and 6 given by the surgeons and surgical residents

	Surgeon total score 0–4	Surgeon total score 5–6
Surgical resident total score 0–4	103	52
Surgical resident total score 5–6	30	98

## Discussion

This study shows a good inter-rater reliability in assessment of the CVS in LC videos between surgeons, considered as the gold standard, and surgical residents. This suggests that surgical residents are proficient in the competency assessment of CVS during an LC. At a vast majority (71% of cases), there was concordance between surgeons and surgical residents in assigning either a score of 0 to 4 or a score of 5 or 6, which is clinically relevant, as the literature defines a satisfactory CVS when the score is 5 or 6 [17]. Within this subgroup (29% of cases) where there was no agreement, surgical residents significantly more frequently assigned a score of 0–4 (63%), suggesting that they appear to score more cautiously than that of experienced surgeons. Fortunately, in the minority (37%) of cases, surgical residents assessed a satisfactory CVS, while surgeons disagreed. These could be the cases in practice where a BDI is likely to occur, highlighting the importance of minimizing such occurrences as much as possible.

Unedited LC videos were utilized in this study for CVS assessment. Existing literature discusses CVS assessment based on intraoperative photographs, of both the anterior and posterior views, offering a cost-effective and easily storable alternative. However, the literature indicates that video recording is still the superior method for accurate CVS assessment [19].

Employing SQA tools to assess the quality of surgical procedures and provide constructive feedback is crucial for enhancing postoperative results. Extensive research has consistently demonstrated the significant influence of surgical quality on clinical outcomes [2, 3, 20–23]. Notably, a national training program called Lapco, implemented in England for specialist colorectal surgeons, successfully employed competency-based supervised clinical training, resulting in reduced mortality and morbidity rates [24]. Applying CVS scoring of LC videos on a larger scale and thus creating valuable feedback opportunities may enable surgeons and especially surgical residents to make faster progress in performing the procedure. This, in turn, may mitigate BDI and positively impact patient outcomes following LC.

One limitation of this study is the absence of comprehensive explanation or training session on how to exactly use the assessment tool and when to assign specific scores. This tool does not pose a significant challenge in terms of usability, but literature suggests that pre-assessment training for the raters could contribute to more consistent results in video-based quality assessment [25]. Despite the tool’s simplicity, providing such training could potentially elevate the ICC scores by fostering consensus among surgeons and surgical residents when assigning scores to specific criteria from the same video.

Scoring videos using assessment tools is inherently time-consuming. As artificial intelligence (AI) gains prominence in the medical field, there is potential for its future integration to facilitate the expeditious determination of the moment of CVS within the entire, unedited LC videos [26]. This would allow reviewers to assess CVS faster. Next, an ideal scenario involves the development of a computer model capable not only of pinpointing the moment of CVS, but also of discerning whether the CVS is deemed “good” and therefore indicating whether the operator can start with ligation of the cystic artery and duct. The literature speaks of a “good” CVS if the score of the 6-point assessment tool is 5 or 6. Training such a model necessitates exposing it to numerous frames of LC videos depicting CVS instances with varying scores. This AI model could eventually be applied intraoperatively, providing a “green” or “red” light post-CVS. In this example, a “green” light would empower the surgical resident to complete the surgery independently, while a “red” light would prompt the surgeon's involvement for guidance. Before such applications become reality, this study establishes that surgical residents can effectively self-assess their surgeries retrospectively and video-based, an essential step in maintaining a steep learning curve.

## Conclusion

Surgical residents are as capable as experienced surgeons in assessing the critical view of safety (CVS) in laparoscopic cholecystectomy (LC) videos. If residents regularly review and assess their own surgical procedures as well as those of others through video analysis, they might learn more quickly and enhance patient care. In addition, incorporating artificial intelligence (AI) into the evaluation of surgical videos in future could accelerate this learning process and potentially even automate certain assessment tasks.
